# Dr. Joseph Lewi

**Published:** 1898-02

**Authors:** 


Dr. Joseph Lewi, of Albany, died at his residence in that city, December 19, 1897, aged 77 years. He was one of the more prominent of the older physicians in that portion of the state and was universally respected by all who knew him. He received his medical education at the University of Vienna and came to this country soon after graduation. He has served as president of the medical society of the county of Albany and at the time of his death was the senior censor of the medical society of the state of New York. He was for twelve years a member of the state board of public instruction and declined reappointment upon the expiration of his fourth term of service. He leaves six sons, two of whom, as well as a son-in-law, Dr. Herman Bendell, are physicians. One daughter died only a week before the decease of her father. He was the father of Dr. Maurice J. Lewi, of New York, secretary of the state board of medical examiners. Dr. Lewi leaves a lasting impression on the professonal and social life of the city of his residence and died lamented by a vast group of relatives, friends and acquaintances.

